# Tissue clearing and 3D imaging in developmental biology

**DOI:** 10.1242/dev.199369

**Published:** 2021-09-30

**Authors:** Alba Vieites-Prado, Nicolas Renier

**Affiliations:** Sorbonne Université, Paris Brain Institute – ICM, INSERM, CNRS, AP-HP, Hôpital de la Pitié Salpêtrière, 75013 Paris, France

**Keywords:** 3D imaging, Light-sheet microscopy, Tissue clearing

## Abstract

Tissue clearing increases the transparency of late developmental stages and enables deep imaging in fixed organisms. Successful implementation of these methodologies requires a good grasp of sample processing, imaging and the possibilities offered by image analysis. In this Primer, we highlight how tissue clearing can revolutionize the histological analysis of developmental processes and we advise on how to implement effective clearing protocols, imaging strategies and analysis methods for developmental biology.

## Abstract (precis)

**Summary:** This Primer offers a practical review of the field of tissue clearing, a process which enables 3D imaging of intact embryos and animals, but can be daunting to implement.

## Introduction

Natural transparency is one of the most desired features of model organisms for developmental studies. It enables the visualization and manipulation of cells *in vivo*, preserving their natural interactions in intact organisms. From the 19th century, the studies of fertilization, zygotic activation, cleavage, gastrulation, neurulation and organogenesis have been performed in a host of model species drawing from the large pool of transparent embryos found in Protostomia, echinoderms, amphibians, teleosts and birds.

Although the early stages of embryogenesis can be amenable to direct observation, as the embryos develop the lipid content, the secretion of fibrous proteins in the extracellular matrix and accumulation of pigment perturb the propagation of light, turning the specimen opaque and, therefore, hampering the study of middle- to late-developmental stages in intact samples. Tissue-clearing techniques aim to re-establish a straight light path through the tissues by partially removing the components that deviate (scatter) or absorb light.

The refractive index, or index of refraction, of a substance indicates how much it delays light propagation in a given medium compared with in a vacuum. The complex composition of biological samples produces a heterogenous refractive index, from 1.33 (water), up to 1.66 (bones), with intermediate indices for proteins and lipids (usually 1.4 to 1.6) (Susaki and Ueda, 2016). This heterogeneity scatters light that, in practice, makes the sample opaque (Richardson and Lichtman, 2015). Reducing the heterogeneity of the refractive indices in a sample is the key concept behind tissue-clearing technology. The aim of these protocols is to first remove compounds with outlier refractive indices and less informative value, usually water, hydroxyapatite (i.e. bone) and lipids, then to bleach pigments, and finally to homogenize the refractive index of left-over compounds.

**Fig. 1.**
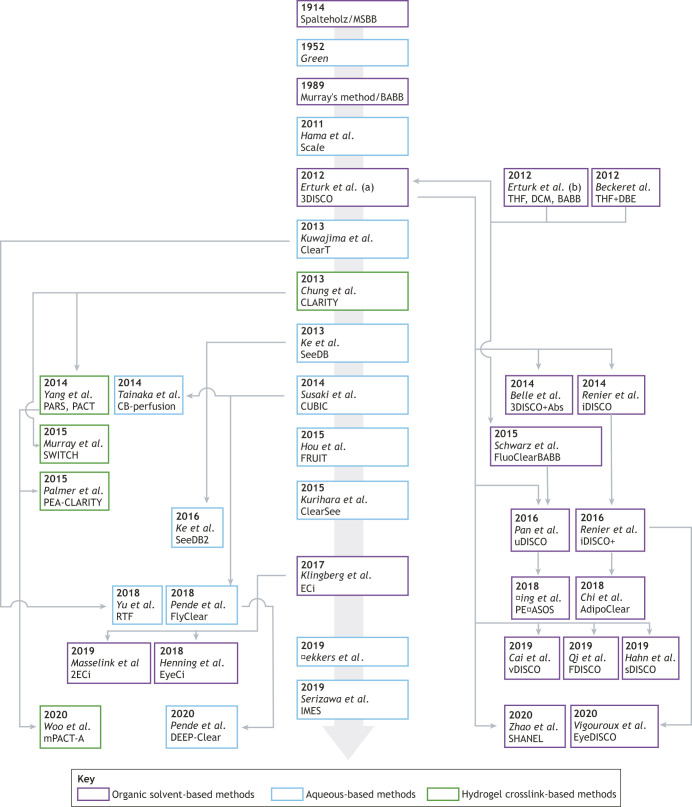
**Genealogy of tissue-clearing methods applied to developmental biology.** The tree illustrates the first publication of each method. Only methods compatible with embryology are listed. Aqueous-based methods (blue); hydrogel crosslink-based methods (green); organic-solvent-based methods (purple). Arrows indicate a derived method. See Table 1 for more details.

Werner Spalteholz developed the first tissue-clearing protocol (Spalteholz, 1911, 1914) using methyl-salicylate and benzyl-benzoate (simplified as MSBB) to turn animals and large organs transparent, enabling the observation of their skeleton and internal organs. In the late 1980s, Andrew Murray modified the method to clarify *Xenopus* eggs, using benzyl-alcohol and benzyl-benzoate (BABB), also known as Murray's method (Dent et al., 1989). This method is the foundation of current organic solvent-based protocols (Fig. 1). Murray's method was combined with bright-field imaging of colorimetric enzymatic reactions or confocal microscopy of fluorescent dyes in cleared embryos of early developmental stages [up to embryonic day (E) 12 in the mouse (e.g. Tischfield et al., 2010)]. These early applications of tissue clearing in embryology enabled a host of studies on the development of the peripheral nervous system or the study of apoptosis during early organogenesis (e.g. Zucker et al., 1998).

**Table 1.**
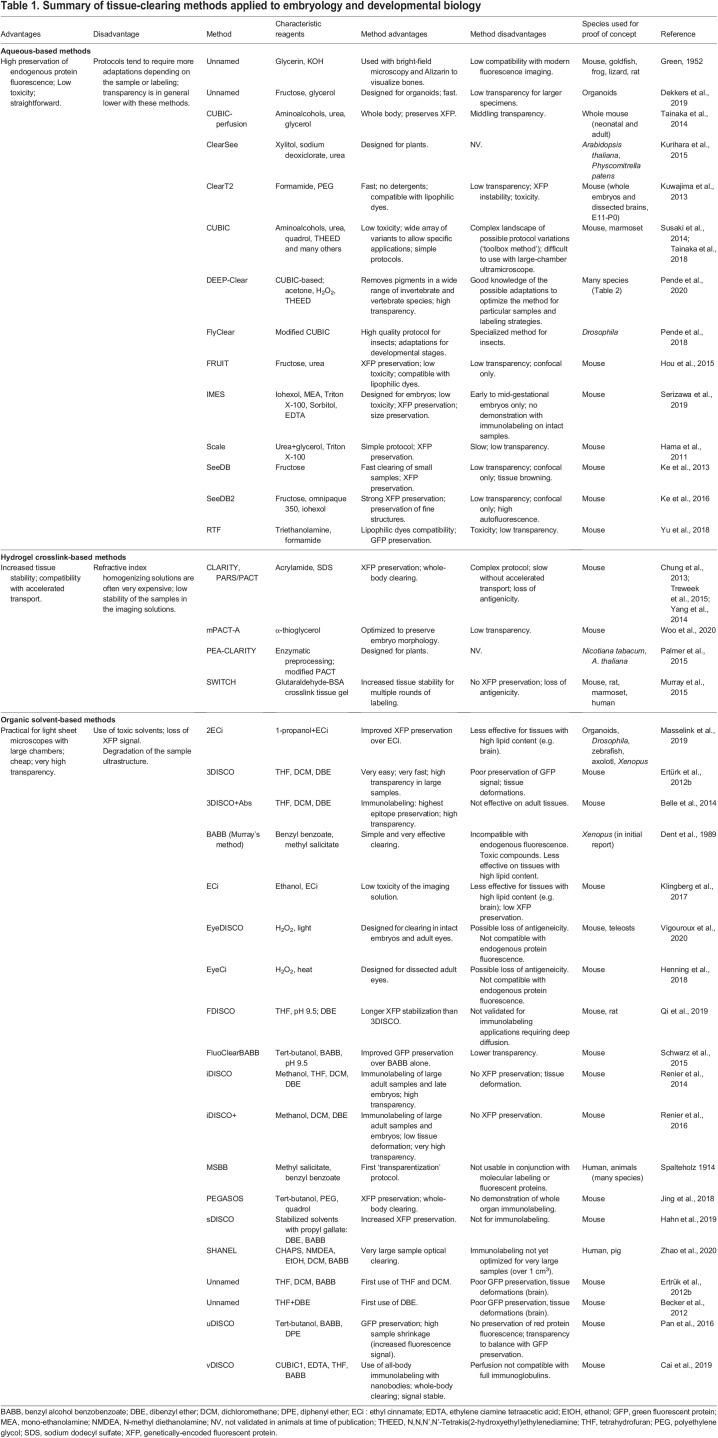
Summary of tissue-clearing methods applied to embryology and developmental biology

In 2007, the combination of light-sheet microscopes (or selective plane illumination microscopes) with tissue clearing on large samples (Dodt et al., 2007) started a race to develop effective tissue-clearing protocols. Light-sheet microscopy offered a gain of speed several orders of magnitude over scanning microscopes and the capacity to image very large samples (over 1 cm); however, it required the development of better tissue-clearing protocols because high transparency is paramount for this imaging modality. Today, this acceleration is enabling studies that could have been considered impossible or too ambitious until recently, such as screening complex phenotypes and inter-organ connections or analyzing the development of intricate 3D structures, such as the vascular and neural networks, the digestive tract, lung or any other tubular system.

Many reviews have discussed tissue-clearing methods, with either an in-depth focus on the physicochemical principles (Richardson and Lichtman, 2015; Tainaka et al., 2016; Yu et al., 2021) or specific applications in neuroscience (Escalante et al., 2020; Ueda et al., 2020a,b; Vigouroux et al., 2017), cardiovascular development (Kolesová et al., 2021), pancreas (Campbell-Thompson and Tang, 2021) or biomedical applications (Almagro et al., 2021; Feuchtinger et al., 2016; Gómez-Gaviro et al., 2020). General guides are also available to help select clearing methods or imaging strategies (Ariel, 2017, 2018; Molbay et al., 2021; Weiss et al., 2021). This Primer aims to point developmental biologists new to tissue clearing in the right direction by first choosing an adequate method. We also highlight the crucial steps of tissue-clearing protocols that can be reused and combined to solve organ or sample-specific challenges in embryology. We then touch on the current possibilities offered by image analysis tools, and finally give inspiring examples of successful applications of tissue clearing in developmental studies.

## Tissue-clearing methods as modular protocols

### The main families of tissue-clearing methods

The currently available methods for tissue clearing are commonly classified in three large families based on the strategy used to homogenize the refractive index across the tissue (Fig. 1). Aqueous methods, such as CUBIC (Susaki et al., 2014), use hydration to help solubilize lipids in micelles, along with hyperosmotic solutions, to raise the refractive index of cellular compartments (usually from 1.3 to ∼1.45). Hydrogel-based methods, such as CLARITY (Chung et al., 2013), increase protein crosslinking to stabilize the structure of the tissue to enable the use of stronger detergents and accelerated transport through the sample. Finally, organic solvent-based methods, such as 3DISCO (Ertürk et al., 2012a), homogenize the refractive index of the sample by dehydration followed by incubation in solvents with a high refractive index (final refractive index ∼1.56), enabling a very high transparency. Regardless of the family, the underlying principle is the same across all methods and they can be combined. The general advantages and pitfalls of these methods are summarized in Table 1.

### Common modules

**Fig. 2.**
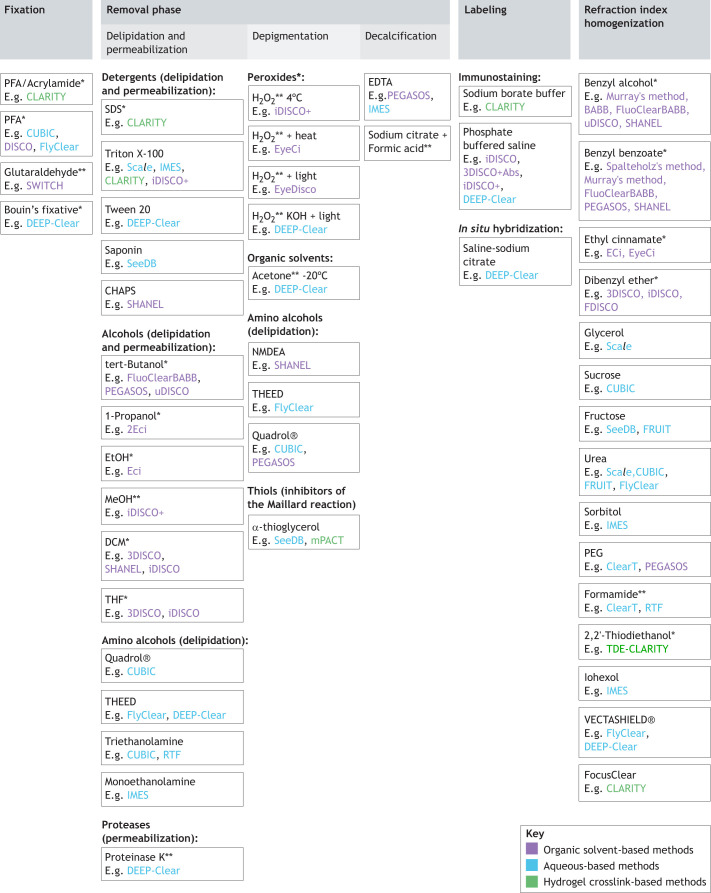
**Common modules used in tissue-clearing methods.** The six modules of tissue-clearing protocols are indicated, with examples of chemicals used in the different protocols. *, reduction or instability of the genetically-encoded protein (XFP) fluorescence signal; **, complete loss of XFP fluorescence; aqueous-based methods (blue); hydrogel crosslink-based methods (green); organic-solvent-based methods (purple).

All tissue-clearing protocols should be understood as modular toolboxes in which specific steps can be added or removed in order to adapt them to specific applications; for example, modules taken from the CUBIC and CLARITY pipelines have recently been combined to clear slices of a rhesus monkey brain (Xu et al., 2021). Most clearing protocols can be broken down to into six modules: fixation, lipid removal or delipidation, decalcification (optional), bleaching or depigmentation/decolorization (optional), labeling (optional) and optical clearing (Fig. 2).

#### Fixation

The fixation module (achieved either with paraformaldehyde alone or combined with additional crosslinkers) maintains the molecular content and structure of the sample along the subsequent processing steps. Underfixation leads to loss of content, whereas overfixation decreases transparency, protein fluorescence and immunoreactivity (Choi et al., 2021).

#### Delipidation

Delipidation is the most crucial part of the protocol because it directly influences transparency, and eventually antibody diffusion. Delipidation is the module for which the diversity of options is the broadest across protocols and the choice of strategy is dictated by whether immunolabeling or endogenous fluorescence are used for the labeling of interest. Fluorescent proteins imaged natively call for special care during delipidation to balance transparency while maintaining the integrity of the protein folding.

#### Optional steps: decalcification, bleaching and labeling

Decalcification and bleaching are optional steps, but bleaching is recommended in most cases to improve tissue background and transparency, even in non-pigmented samples. The labeling step entails the use of fluorescent probes or immunoglobulins to label structures of interest.

#### Optical clearing

Finally, the optical clearing module is the stage at which the refractive index is homogenized to achieve optical transparency. For this module, one needs to take into account the microscope and objectives used, the necessity to preserve (or not preserve) protein fluorescence, the expansion or shrinkage of the sample and transparency requirements.

## Choosing a tissue-clearing method

To choose a family of methods, one should consider the microscope that will be used, the nature of the signal and the sample size and age.

### Scanning or light-sheet microscopy?

The type of microscope used for imaging may dictate the initial choice of protocol depending on the resolution required, and the size and type of sample chamber available.

#### Scanning microscopes

Scanning microscopes (e.g. confocal or two-photon) are the best choice for studies requiring precise signal colocalization and high resolution on smaller samples, thanks to the availability of high-quality corrected optics and the co-linearity of the illumination and imaging light paths. As the light comes in and out of the sample from the same side, and thanks to the shallow focal plane obtained from the pinhole, it is possible to use scanning microscopes in small samples with any tissue-clearing method – even those that have poorer optical clearing capabilities. Therefore, these microscopes combine well with protocols that clear less efficiently but offer the best fluorescence preservation, such as SeeDB2 (Ke et al., 2016). SeeDB2 also has the advantage of allowing a fine tuning of the refractive index in the mounting solution to match the lens correction, enabling multicolor, high-resolution imaging (Ke et al., 2016). There are two important parameters to consider when choosing a method for confocal imaging. (1) The sample has to be housed in a vessel compatible with the objective used; either a multiwell plate for inverted microscopes or an imaging chamber for upright systems. Multiwell plates enable the scanning of multiple samples at once, but holding the samples in place is challenging. Non-binding epoxy sealants can help secure the sample. (2) The refractive index has to be consistent across the light path, which includes the mounting solution, coverslip glass and lens correction collar. Using specialized glass imaging chambers, or building a custom chamber from coverslips and spacers, is necessary to ensure the best optical resolution. Therefore, oil-corrected lenses (1.5) would perfectly match standard coverslips and the organic solvents found in 3DISCO (DBE; Ertürk et al., 2012a) or ethyl cinnamate (ECi; Klingberg et al., 2017), whereas CUBIC or CLARITY would call for lenses optimized at 1.45.

#### Light-sheet microscopes

**Fig. 3.**
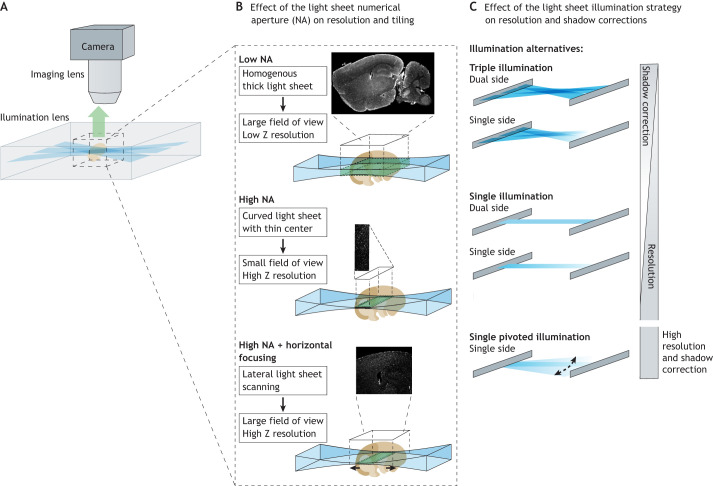
**Optimization of light-sheet microscopy for embryology: maximizing the resolution.** (A) Schematic of light-sheet illumination and light collection (example of the ultramicroscope). (B) Importance of the numerical aperture (NA) for the light-sheet generation and its effects on the homogeneity of the optical plane and the size of the field of view. Examples are given with vascular labeling: at low axial resolution, the vessels appear continuous, whereas at high resolution they are shown with their cross-sections as points. (C) Types of illumination. Ultramicroscopes incorporate multiple angles or dual-side illumination to reduce the shadows and improve the illumination width in large samples. The MesoSPIM uses dual-sided illumination and horizontal scanning to speed up the system efficiently. Finally, the Zeiss systems use a pivoted illumination system to reduce the shadows and incorporate a mechanical arm to rotate the sample.

Tissue clearing enables the use of light-sheet microscopy on centimeter-sized samples. Light-sheet microscopes generate a thin plane of light through the sample, while an orthogonal imaging objective collects the image from the illuminated plane (Fig. 3). Light-sheet microscopes with large open sample chambers can scan bigger samples fast, but require high transparencies. Therefore, they best combine with organic solvent-based methods, such as 3DISCO or iDISCO (Belle et al., 2014; Renier et al., 2014) . Indeed, organic solvent-based imaging solutions are cheaper and more stable in large volumes than aqueous clearing solutions, for which evaporation can cause imaging artefacts (Table 1). The ultramicroscope design is the workhorse of such large-sample imaging, up to a few cubic centimeters in size. Typical magnifications on commercial systems are relatively low, ranging between 1× and 12× (5-0.5 µm pixel size) on the Miltenyi UM2 or Blaze, while the light sheet thickness is constant at a maximum optical resolution of 4 µm (Fig. 3). Light-sheet systems using smaller closed chambers, such as spectrophotometer cuvettes [e.g. the Mesospim (Voigt et al., 2019) or the Zeiss Z7] prevent the evaporation of the imaging solution, and thus allow for more a flexible use of diverse tissue-clearing protocols and ease sample mounting. They are, however, more restricted regarding sample sizes. These systems are the best all-purpose choice, especially when endogenous fluorescence imaging of larger volumes is required, and combine with CUBIC, CLARITY as with solvent-based protocols. Unfortunately, most of these designs are not commercial and have to be built and maintained by the users. Even when maximizing the attainable resolution, 3D imaging still produces relatively low-resolution images compared with sections from epifluorescence or confocal microscopes.

### Fluorescent proteins or immunolabeling?

Second to the imaging microscope, the source of the fluorescent signal is the most crucial parameter when choosing a method. An essential consideration to optimize a protocol is the balance between transparency and the signal obtained from molecular probes or fluorescent proteins. High transparency comes with more stringent tissue processing, which is detrimental to the quality of labeling, either by affecting antigenicity or the brightness of a fluorescence signal. Below, we suggest some of the families best suited for different sources of fluorescent signal and we discuss considerations for optimizing specific modules in the next section.

#### Fluorescent proteins

Organic solvent-based imaging solutions are often difficult to combine with the preservation of protein fluorescence. Instead, aqueous-based methods, such as CUBIC and their derivatives, are well-suited to imaging fluorescent proteins. However, delipidation, permeabilization and bleaching affect fluorescent protein detection (discussed in the next section). Therefore, the imaging of endogenous fluorescence should be restricted to highly expressed transgenes.

#### Immunolabeling

When whole-mount immunolabeling is needed, an organic solvent-based protocol (e.g. 3DISCO/iDISCO+) is a good starting point, because it provides a practical framework for immunolabeling that resembles traditional histology. Unlike fluorescent proteins, most organic fluorophores, such as AlexaFluor^©^ or DyLight^©^, are compatible with organic solvent-based protocols (Belle et al., 2014; Renier et al., 2014). Therefore, immunolabeling or other tagging methods using these conjugates allow for more flexibility for optical clearing than fluorescent proteins.

Hydrogel-based methods, such as CLARITY (Chung et al., 2013), provide tissue stabilization that is well suited for applications requiring multiple rounds of immunolabeling. The stabilization also enables active transport of antibodies or probes via physicochemical forces to speed up diffusion (Kim et al., 2015; Ku et al., 2020; Park et al., 2018; Yun et al., 2019 preprint). Certain variants of CUBIC, such as CUBIC-HV are also compatible with immunolabeling, but may require specific optimizations (temperature, blocking and detergents) for different primary antibodies (see below) (Susaki et al., 2020).

### Sample type

The sample itself must also be considered when choosing a protocol. The success of the method will depend on the biochemical complexity and the size of the specimen. 

#### Sample stage

Clearing protocols are primarily designed for adult tissues, but most are transferrable to embryonic tissues with minor adaptations. Of note, ClearT (Kuwajima et al., 2013), mPACT-A (Woo et al., 2020) and 3DISCO+Abs (Belle et al., 2014) are among the few protocols primarily developed for developmental stages (up to the first postnatal week in the mouse). Some qualities of tissue clearing, such as transparency, tissue autofluorescence and antibody diffusion, are better in embryos over adult samples, due to the reduced presence of complex lipids (triglycerides, ceramides, sphingolipids), reduced glycosylation, calcification, hairs, cell adherence and absence of lipofuscin. However, the fragility and heterogeneity of embryos may require adaptations when using tissue-clearing protocols primarily designed for adult samples.

#### Sample size

Embryos can span an extreme range of sizes, from sub-millimeter to several centimeters. Embryo size can be manipulated, to a certain extent, to facilitate imaging. For example, expansion of small samples improves the spatial resolution (Chen et al., 2015; Wassie et al., 2019). On the other hand, shrinking large samples facilitates imaging, either when the specimen cannot fit in the imaging chamber or as a strategy to boost the fluorescence signal by increasing the volume concentration of dyes. The use of phosphate-buffered saline (PBS) diluted in methanol (instead of water in methanol) during the dehydration steps of organic solvent-based protocols (Pan et al., 2016; Renier et al., 2016) reduces the sample size (Table 1). For CLARITY-based protocols (Table 1; Fig. 1), the hydrogel density and the homogenization solution chemistry both modulate the sample size (Treweek et al., 2015). Small embryos can be challenging to handle, so adding an inclusion step in agarose gel before dehydration in solvent-based methods is a good optional step to facilitate their manipulation.

#### Plants

Finally, although plants are not the main focus of this Primer, tissue clearing is also used in vegetal tissues, in which the cellulose of the cell wall and plant pigments present specific challenges. A modified protocol based on Hoyer's solution was used to clarify *Arabidopsis thaliana* embryos (Bougourd et al., 2000) until the adaptation of CLARITY to plants (PEA-Clarity; Fig. 1) by incorporating an enzymatic digestion step targeting the cellulose wall (Palmer et al., 2015). ClearSee (Fig. 1) is a solution specially designed for plants, preserving the signal of genetically-encoded fluorescent proteins (XFP) while reducing the autofluorescence of chlorophyll. DEEP-clear, originally developed for animal pigments (Pende et al., 2020), also quenches chlorophyll fluorescence. ClearSee uses xylitol, sodium deoxycholate, urea and water as its main components and was first tested in seedlings, leaves and pistils of *A. thaliana*, and moss gametophores (Kurihara et al., 2015).

## Customizing and troubleshooting a tissue-clearing method

**Fig. 4.**
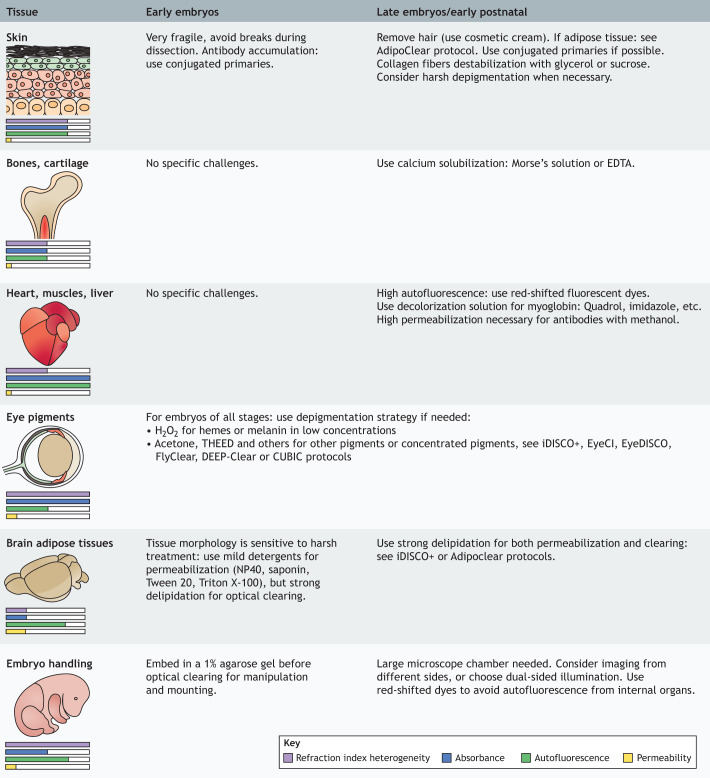
**Possible optimization of tissue**-**clearing protocols in early and late embryos.** Standard protocols should be followed by default, but in some situations, optimizations are needed to reveal a weak signal or difficult staining. This figure provides some suggestions for possible modifications to enhance the signal of a few complex organs.

The following paragraphs suggest possible adaptations to protocol steps to address specific challenges associated with different organs (Fig. 4) and fluorescent signals (mentioned above). Tainaka and colleagues have also proposed a comprehensive guide to the compounds that can alter the properties of tissue clearing (Tainaka et al., 2018). Even though it is applied here to CUBIC methods, it can also be mixed and matched with other protocols to improve specific properties of the cleared tissue.

### Delipidation

As mentioned previously, delipidation is an essential step (Fig. 2) to achieve transparency but is also crucial for the permeabilization of the tissue when immunolabeling is needed. Adult tissues require the use of harsh detergents (e.g. high concentrations of SDS) or solvents for complete permeabilization. However, mild delipidation can suffice for immunolabeling in embryos, with a more substantial delipidation at the end of the protocol to achieve high transparency. The use of methanol, enabling both delipidation and permeabilization, can prevent some epitopes from being recognized but can also unmask hidden epitopes, so comparing antibody signals in a methanol-free versus methanol-based protocol is a valuable optimization (Renier et al., 2014). Often, embryos do not require methanol treatment for permeabilization up to postnatal day (P) 7 in the mouse brain (Fig. 4). The Adipoclear modification of iDISCO+ improves delipidation for developed adipose tissues that accumulate triglycerides (Chi et al., 2018). It is necessary to adjust the intensity of the permeabilization step in younger embryos because excessive delipidation can damage the morphology of lipid-rich organs, such as the brain, while protein-rich organs, such as muscles or the liver are less affected. Detergents found in the 3DISCO or iDISCO (no methanol) methods, such as NP-40, Tween-20, Triton X-100 or Saponin, are all indicated for the permeabilization of embryos (up to P7 in the mouse brain) instead of solvents. CUBIC protocols employ other permeabilization strategies with amino-alcohols optimized for different samples and applications, as well as more recently sodium dodecylbenesulfonate or Triton X-100. A list of possible aminoalcohols used in CUBIC can be found in Fig. 2 and Tainaka et al., 2018.

Delipidation will usually prevent lipophilic-labeling strategies popular in developing embryos, such as carbocyanine dyes (e.g. DiI). Detergent-free protocols, such as ClearT or Fruit, are specifically compatible with lipophilic tracers but have low transparency (Hou et al., 2015; Kuwajima et al., 2013).

Delipidation with solvents is difficult to combine with the preservation of protein fluorescence, but not impossible. XFP are susceptible to dehydration in short hydrocarbon-chain alcohols, such as methanol, which are the most effective at solubilizing lipids (Schwarz et al., 2015). However, longer-branched alcohols, such as tert-butanol, are very good at preserving GFP fluorescence, but their delipidating action is usually slow (Jing et al., 2018; Pan et al., 2016) and they do not preserve the fluorescence of most other families of fluorescent proteins, such as tdTomato (Qi et al., 2019). Additives, such as diphenyl ether, vitamin E or polyethylene glycol (PEG), dissolved in organic solvents can help preserve fluorescence, usually at the expense of transparency. High pHs are also an effective way to protect fluorescence (Hahn et al., 2019; Jing et al., 2018; Pan et al., 2016; Qi et al., 2019; Schwarz et al., 2015). XFP fluorescence is less of an issue with aqueous clearing protocols, but the delipidation must be optimized to achieve good transparency while preserving the signal.

### Bleaching/depigmentation

Pigments are commonly present in the skin, visual organs and exoskeletons of animals. This includes, for example, the hemoglobin of erythrocytes, the hemocyanin of the invertebrate hemolymph, the myoglobin in the muscle cells, as well as melanin in the skin, hair or retina. Invertebrates can synthetize additional pigments, such as astaxanthin, ommochromes and pterins (present in the wings of butterflies). Most clearing protocols include a general step of bleaching/depigmentation that consists of incubation of the sample with a low concentration of H_2_O_2_ (3-6%). This step is usually sufficient to remove small amounts of hemoglobin, myoglobin and melanin. However, it is not effective against high concentrations of melanin, such as those found in hair or the retina, or the pigments of most invertebrate species – although it can work in crustaceans and amphibians (Konno and Okazaki, 2018; Masselink et al., 2019). EyeCI and EyeDISCO address the challenge of retinal pigments in the dissected eye of adult or embryonic mice by combining H_2_O_2_ with heat or light inactivation (Henning et al., 2018; Vigouroux et al., 2020). H_2_O_2_ used in an aqueous solution at room temperature has the drawback of generating air bubbles that can damage, and stay trapped in, the sample, blocking the light path. Pende and colleagues tackled the problem of depigmentation broadly by offering a toolbox of reagents, from FlyClear to DEEP-Clear (Fig. 1), a depigmentation procedure that showed effective discoloration of adult annelids, cephalopods, teleostean and amphibian using a modified CUBIC solution (Pende et al., 2018, 2020). Depigmentation treatments, such as H_2_O_2_, THEED or acetone, quench endogenous fluorescent reporters. Decolorization treatments found in CUBIC protocols and others, such as Quadrol, are usually compatible with the preservation of endogenous fluorescence.

### Decalcification

Calcified structures constitute a significant obstacle for imaging due to their high refractive index. To overcome this issue, decalcification steps (Fig. 2) can be added to established protocols. Decalcification is achieved either with Morse's solution (Jacob et al., 2019) or with EDTA (Jing et al., 2018; Konno and Okazaki, 2018; Perin et al., 2019). Morse's solution (1:1 solution of 20% trisodium citrate and 45% formic acid) is much faster (in the order of minutes), but can be detrimental to the tissue and antigenicity, while EDTA is slow (days to weeks), but gives better results.

### Labeling

Antibodies can diffuse deep (over 1 cm) in embryos and young tissues. The strong delipidation present in some protocols, such as CLARITY, CUBIC (in some variants) or iDISCO+ enable the deep diffusion of these probes. Therefore, whole-mount labeling of large, intact samples works well hand in hand with tissue clearing. However, the following points must be optimized. (1) When antibodies are used too concentrated, they accumulate at the surface of the sample. In rare examples, low amounts of antibody molecules can lead to depletion; therefore, antibody dilution from the stock must be optimized. (2) If staining for a dense epitope, using conjugated primary antibodies instead of secondary amplification can often solve diffusion issues at the expense of an overall lower signal (Susaki et al., 2020). Using conjugated primaries (i.e. bypassing the secondary) also solves the presence of antibody precipitates at the embryo skin surface or in large blood vessels. (3) Finally, longer incubation times improve antibody diffusion, but the gains in signal homogeneity are obtained non-linearly, so compromises should often be made between protocol length and deep signal. Incubation time should be adapted to the developmental stage, from 24 h (for samples <E12 in mice) to 1 week (newborns). Of note, owing to the long incubations and permeabilization steps, immunolabeling often is not compatible with the preservation of native protein fluorescence. In our experience, nanobodies usually yield low signal, but have been efficiently used for whole-body perfusion to boost the signal of endogenous tags, such as GFP or RFP (Cai et al., 2019; Pan et al., 2019).

## Analysis of cleared samples

The analysis of data obtained from light-sheet or confocal microscopes on cleared tissue is a daunting task that often represents most of the person-hours of these experiments. A mid-gestational mouse embryo (about 1 cm^3^) scanned at a 5 µm resolution (about 1× magnification) uses about 10 GB of uncompressed image data for each channel. The size increases to about 200 GB at a 1.6 µm resolution (about a 4× magnification). 3D imaging data impose challenges to store, move and display the data requiring good pipelines from the microscope to the experimenter's desk.

### Stitching tiled acquisitions

Often the sample is not captured in its entirety within the field of view. It is therefore often necessary to tile the scan across a grid of adjacent stacks. Tiled acquisitions require an image stitching step, to align and fuse adjacent stacks to reconstruct the full image. This is a complex operation; good image positioning and blending are crucial to insure a seamless 3D image devoid of striping or duplication effects. Imaris (Oxford Instruments) and Vision 4D (Arivis) offer basic stitching tools that scale well to large data sizes. The free software, TeraStitcher (Bria and Iannello, 2012) is also a fast stitcher for large data. However, the perfect stitching of a mosaic often calls for specific corrections for chromatic shifts, stage movements and spherical aberrations; BigStitcher is a suite of tools that addresses specific challenges of stitching data obtained from cleared samples (Hörl et al., 2019). Finally, our WobblyStitcher addresses specific challenges of stage movement problems by providing a tracing-based correction of the microscope's erratic movements during the acquisition (Kirst et al., 2020).

### Data visualization

**Fig. 5.**
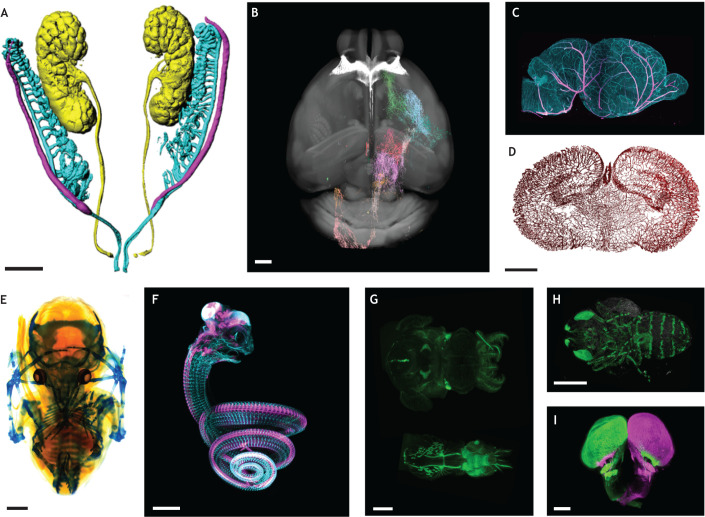
**Examples of applications of tissue clearing to developmental biology.** (A-D) Examples of image analysis. (A) Imaris surface segmentation of a GW8 human embryo, showing the urogenital system. This type of semi-automated segmentation allows researchers to highlight structures of interest, even in complex or noisy data (Belle et al., 2017). (B) Automated axon segmentation of cortico-fugal projections of the barrel cortex with TrailMap, annotated to the mouse Allen Brain Atlas template (https://allensdk.readthedocs.io/en/latest/reference_space.html) with the regional color code of the Allen Brain Atlas. The brain reference template is shown in gray for orientation. (C) 3D view and sagittal projection (300 µm thickness) of a P2 mouse brain stained for the vasculature [CD31/podocalyxin (blue); Sm22 (pink)] with iDISCO+. (D) Coronal slice of the vascular graph from C obtained through an automated segmentation using TubeMap (ClearMap2). Blood vessels were automatically segmented from the 3D scan obtained in C, and embedded into a graph representation, with vessels coded as edges and bifurcations as nodes. The image shows a 3D coronal slice through the reconstructed graph, which reveals the orientations and densities of vessels across different brain regions. Images in B-D kindly provided by Grace Houser and Elisa de Launoit (Paris Brain Institute, France; unpublished). (E-I) Applications of tissue clearing to evo-devo studies. (E) Short-tailed fruit bat (*Carollia perspicillata*) in developmental stage 19, stained with Alcian Blue and cleared with BABB, imaged with a bright-field stereomicroscope. Image kindly provided by Idoia Quintana-Urzainqui, Paola Bertucci, Peter Warth, Chi-Kuo Hu and Richard Behringer (University of Texas MD Anderson Cancer Center, TX, USA; unpublished). (F) 3D view of an African house snake embryo (8 days post oviposition) stained for Robo3 (red) and βIII-Tub (green) with 3DISCO and imaged with a light-sheet ultramicroscope. Image kindly provided by Alain Chédotal (The Vision Institute, France), produced as described in Friocourt et al. (2019). (G) Longfin inshore squid and Hawaiian bobtail squid stained for acetylated tubulin with the DEEPClear protocol. Adapted from Pende et al. (2020). (H) Fruit fly (*Drosophila melanogaster*) pupa expressing GFP in sensory neurons, cleared with FlyClear (Pende et al., 2018). Images (G,H) kindly provided by Marco Pende (Vienna University of Technology, Austria). (I) 3D light-sheet image of the brain of a spotted gar injected in the eyes with two cholera toxin β tracers (Vigouroux et al., 2021). Scale bars: 400 µm.

The data visualization is best carried out with software using pyramidal dynamic rendering, in which the resolution of the data displayed changes automatically with the zoom, such as Imaris (Oxford Instruments) or the Virtual-Reality only SyGlass (IstoVisio). A vital function for data visualization is the capability to quickly draw 3D arbitrary volumes to highlight regions of interest or carve out signals from an unwanted region (Fig. 5A). This can be done plane-wise (e.g. with Imaris or Vision4D) or with virtual reality (SyGlass).

### Quantification: segmentation

Automated object segmentation, the capacity of an algorithm to recognize voxels in an image belonging to a structure (axons, vessels, tubes etc.) or cells of interest, is also an essential aspect of quantitative analysis. Commercial software offers several tools ready out-of-the-box to segment cells, axons, vessels and others. These make use of either tracing methods or parametric filters, which do not scale well to large data, so often have to be used on a small crop of the data. Vision 4D integrates learning-based segmentation from Ilastik using Random Tree Forest classifiers, which can scale to large data and is very fast to train (Berg et al., 2019; Sommer et al., 2011). However, this strategy only works on simpler tasks when the quality of the background and objects are relatively homogenous. Convolutional neuronal networks have been recently used successfully in different complex segmentation tasks applied to cleared samples (Fig. 5B-D) (Friedmann et al., 2020; Kirst et al., 2020; Pan et al., 2019; Todorov et al., 2020). They have two advantages: they can solve highly complex segmentation tasks and they scale well to the considerable size of the data. However, they have two weaknesses: their training is extremely slow and labor-intensive and they rarely work well outside of a narrow definition of the type of data they were trained for (reviewed by Hallou et al., 2021). Often, specific tasks of image analysis require custom pipelines, which are now regularly published (Jin et al., 2019 preprint; Renier et al., 2016; Susaki et al., 2020; Wang et al., 2021; Young et al., 2020), but require the use of scripts and user interventions on the source code, putting them out of reach of most users. Building user-friendly analysis pipelines is an urgent endeavor for this field.

### Registration

In order to facilitate statistical comparisons between samples, 3D datasets can be aligned either to each other or to a reference template. A reference template enables the overlay of an anatomical annotation of the structures. This is essential to obtain automated region-based statistics of the distribution of cells, vessels or axons (Friedmann et al., 2020; Kirst et al., 2020; Renier et al., 2016). While the registration of adult samples is relatively straightforward, embryos develop quickly and reference templates are usually not available for them. For the mouse brain, for example, very precise atlases are available for adults (Wang et al., 2020). As generating standard atlases is a labor-intensive task, developmental atlases have been generated for only a few developmental stages (Young et al., 2021). Moreover, developmental times can differ widely between embryos of the same litter, preventing their direct alignment. Manual 3D alignment is possible with Imaris and automated alignment with Elastix (Shamonin et al., 2014) or BIRD (Wang et al., 2021), but the next frontier would be to develop registration tools that can cater specifically to the challenges of embryology: high deformability of the samples, developmental heterochronies and strong anatomical differences between developmental stages necessitating the generation of templates and atlases for each age.

## The broad potential of tissue clearing for developmental biology

Here, we explore the advantages of tissue clearing for comparative developmental anatomy, the study of organs and systems, and unbiased quantifications. We first focus on applications that would not have been possible with standard histology, and then broaden our survey to highlight the diversity of organs and embryos that have been processed with tissue clearing.

### Evolutionary developmental biology (evo-devo)

The universal biochemical makeup of living organisms often enables straightforward reuse of published protocols to different species. Thanks to depigmentation and decalcification, as well as the possibility of clearing samples of any size, tissue clearing can be a boon for evo-devo studies because it can be used on any species irrelevant of their phylogenetic position. The development of clearing methods first focused on clarifying the mouse brain and embryos, but quickly expanded to whole-body postnatal mice (Table 1). Beyond these initial applications, tissue clearing has been successfully tested in most phyla (Table 2), including human embryonic (Belle et al., 2017) and adult (Zhao et al., 2020) tissues. Tissue clearing can also accelerate comparative studies of the organization of complex processes during development (Fig. 5E-I). Comparative studies have used tissue clearing to study the expression of axon guidance receptors in amniotes (Friocourt and Chédotal, 2016; Friocourt et al., 2017, 2019) (Fig. 5F), the development of the peripheral nervous system in a host of different phyla (Pende et al., 2018, 2020) (Fig. 5G,H), as well as the emergence of binocular vision in vertebrates (Vigouroux et al., 2021) (Fig. 5I).

**Table 2.**
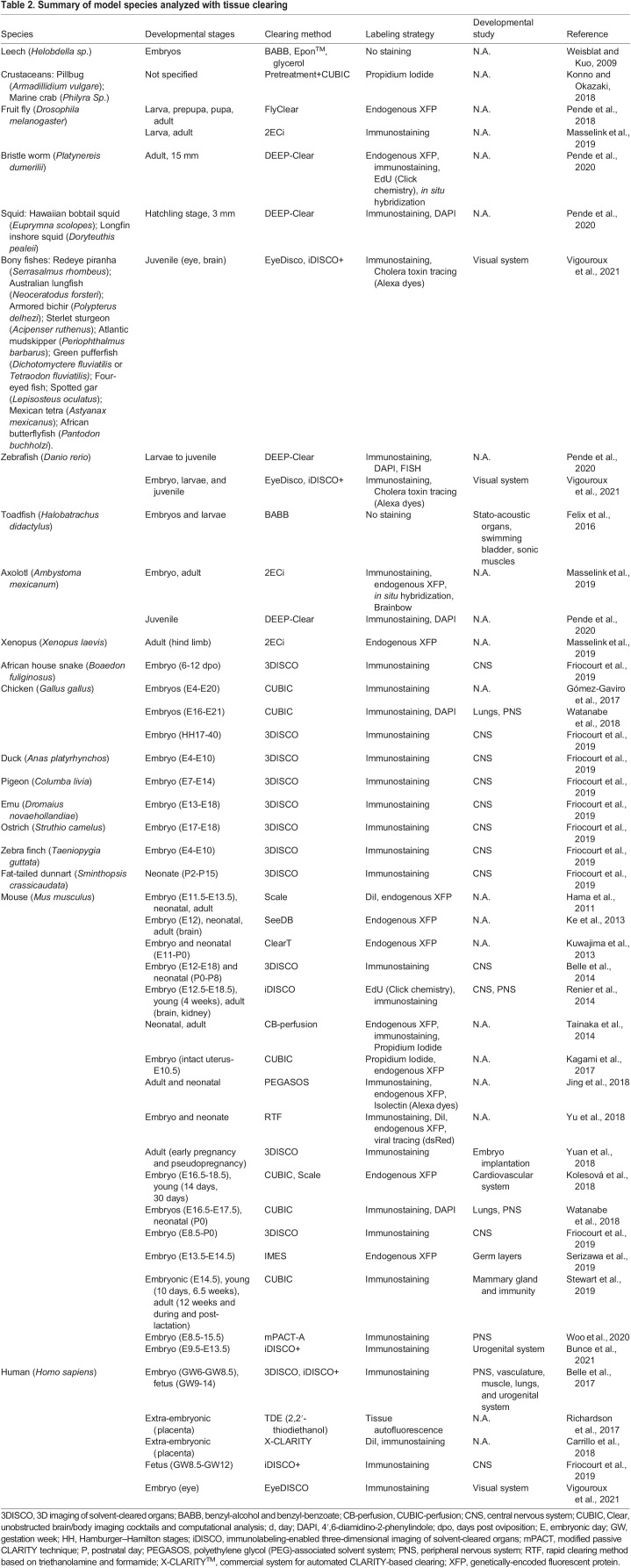
Summary of model species analyzed with tissue clearing

### Qualitative studies of the development of whole organs and systems

Tissue clearing can help to parse the development of complex organs. It is rare for developmental processes to fully localize on a plane or be compatible with open-book preparations. For example, in the central nervous system, mice that have mutations in the genes that encode for the axon guidance molecules netrin 1 (a secreted guidance molecule) and DCC (a netrin 1 receptor) have axon guidance defects in the projection connecting the pons with the habenula, but the looping trajectories of the axons in these mutants made it difficult to analyze the phenotypes from sections alone (Schmidt et al., 2014). Using 3DISCO tissue clearing, it could be shown that the axon guidance defects are different in netrin 1 compared with DCC mutants, suggesting the presence of additional netrin 1 receptors (Belle et al., 2014).

3D imaging is also extremely valuable in studying the interaction of embryos with their extra-embryonic tissues. For example, Yuan and colleagues have used 3DISCO tissue clearing and 3D imaging to study embryo implantation in the mouse (Yuan et al., 2018). 3D imaging has enabled them to survey the complete topography of glands within the uterine epithelium and document how the evagination of the luminal epithelium organizes a complex 3D array of glands that are essential for the implantation and interaction with the blastocyst. The direct apposition and the geometry of the glands inside the crypts would have been very arduous to disentangle from thin sections. Indeed, the development of a wide range of organs has been described in 3D (Table 1).

Tissue clearing is also an essential asset for *in vitro* 3D models of development, such as organoids, because each sample develops in a unique way (i.e. forming internal cavities). It is impossible *a priori* to know how to slice the sample to visualize the cell layers. Moreover, it is difficult to know precisely how many cavities an organoid has from 2D sections. For example, Birey and colleagues have used iDISCO to clear cerebral spheroids and assess GABAergic neuron saltatory migration (Birey et al., 2017). In addition, Masselink and colleagues have demonstrated the suitability of the ECi-based protocol to clarify human cerebral organoids (Masselink et al., 2019), and Dekkers and colleagues have designed an aqueous method based on fructose and glycerol able to render a wide array of organoids transparent, including human intestinal, colonic, breast tumor, fetal liver and airway organoids, as well as murine mammary gland organoids (Dekkers et al., 2019).

### Quantitative studies

Embryos, larvae or young can be imaged whole, enabling a system's view of the process studied, opening up the possibility to align entire organisms to compare different samples. Imaging large fields of views also enable a statistical view of discrete effects by registering several cases to the reference template. A typical application of this technology is quantifying cell location in the brain (Murakami et al., 2018) or immediate early gene expression induced by neuronal activity (Renier et al., 2016; Susaki et al., 2015; Ye et al., 2016). The use of accurate quantifications in 3D images has enabled the detection of subtle, but significant, changes in the vasculature topology following a loss of auditory sensory inputs in congenitally deaf mice lacking the otoferlin gene (Kirst et al., 2020), which has shed light on the importance of sensory inputs to shape the postnatal developmental plasticity of the vascular system at all auditory relays.

## State-of-the-art and future perspectives

3D analysis of biological tissues with tissue clearing is a reinvention of classic 2D histology. It enables an unbiased sampling of histological information that has the potential to empower biologists to design ambitious screens for novel developmental phenotypes, akin to how RNA-sequencing transformed our approach to molecular screening. There are challenges hindering today's broader adoption of tissue clearing, as developing novel assays is slow and requires mastering and optimizing the elements of a complex experimental chain (histological processing, imaging and computational analysis). However, as more and more applications are published, the barrier to entry lowers. Comparative developmental studies and mutant phenotypic screens stand to gain the most from the streamlining of 3D imaging offered by modern tissue clearing and light-sheet microscopy, on top of the reconstructions of complex 3D organs or systems.

Although we have documented here a few successful applications of tissue clearing to developmental biology, there is room for improvement to bring 3D imaging on-par with what is possible with traditional histology. 3D imaging suffers from relatively low imaging resolutions, hampering the analysis of sub-cellular or cellular processes. It would be challenging currently to image cellular morphologies, such as vascular tip cells, migrating neurons or inter-cellular interactions, in large 3D volumes (around 1 cm^3^). Therefore, innovations in large-scale light-sheet microscopy are needed to enable novel applications for tissue clearing and light-sheet imaging, which can be applied to developmental biology and bridge the resolution gap with thin-section histology. Higher resolution images will require better solutions for data handling and analysis, both in terms of performance as well as accessibility to non-expert users. Sample registration (discussed above) is a major challenge for developmental biology and requires the development of dedicated alignment tools.

However, thanks to the very active community developing tissue clearing, our hope is that the wide adoption of these methods will drive the design of unbiased imaging studies that will likely foster many unexpected and serendipitous discoveries.

## References

[DEV199369C1] Almagro, J., Messal, H. A., Thin, M. Z., Van Rheenen, J. and Behrens, A. (2021). Tissue clearing to examine tumour complexity in three dimensions. *Nat. Rev. Cancer* [Epub ahead of print]. 10.1038/s41568-021-00382-w 34331034

[DEV199369C2] Ariel, P. (2017). A beginner's guide to tissue clearing. *Int. J. Biochem. Cell Biol.* 84, 35-39. 10.1016/j.biocel.2016.12.009 28082099 PMC5336404

[DEV199369C3] Ariel, P. (2018). UltraMicroscope II – A User Guide [Internet]. Chapel Hill, NC: University of North Carolina at Chapel Hill. 10.17615/C69M15 30689332

[DEV199369C4] Belle, M., Godefroy, D., Dominici, C., Heitz-Marchaland, C., Zelina, P., Hellal, F., Bradke, F. and Chédotal, A. (2014). A simple method for 3D analysis of immunolabeled axonal tracts in a transparent nervous system. *Cell Reports* 9, 1191-1201. 10.1016/j.celrep.2014.10.037 25456121

[DEV199369C5] Belle, M., Godefroy, D., Couly, G., Malone, S. A., Collier, F., Giacobini, P. and Chédotal, A. (2017). Tridimensional visualization and analysis of early human development. *Cell* 169, 161-173.e12. 10.1016/j.cell.2017.03.008 28340341

[DEV199369C6] Becker, K., Jährling, N., Saghafi, S., Weiler, R. and Dodt, H. U. (2012). Chemical clearing and dehydration of GFP expressing mouse brains. *PLoS ONE* 7: e33916. 10.1371/journal.pone.0033916 22479475 PMC3316521

[DEV199369C7] Berg, S., Kutra, D., Kroeger, T., Straehle, C. N., Kausler, B. X., Haubold, C., Schiegg, M., Ales, J., Beier, T., Rudy, M. et al. (2019). ilastik: interactive machine learning for (bio)image analysis. *Nat. Methods* 16, 1226-1232. 10.1038/s41592-019-0582-9 31570887

[DEV199369C8] Birey, F., Andersen, J., Makinson, C. D., Islam, S., Wei, W., Huber, N., Fan, H. C., Metzler, K. R. C., Panagiotakos, G., Thom, N. et al. (2017). Assembly of functionally integrated human forebrain spheroids. *Nature* 545, 54-59. 10.1038/nature22330 28445465 PMC5805137

[DEV199369C9] Bougourd, S., Marrison, J. and Haseloff, J. (2000). An aniline blue staining procedure for confocal microscopy and 3D imaging of normal and perturbed cellular phenotypes in mature Arabidopsis embryos. *Plant J.* 24, 543-550. 10.1046/j.1365-313x.2000.00892.x 11115135

[DEV199369C10] Bria, A. and Iannello, G. (2012). TeraStitcher - a tool for fast automatic 3D-stitching of teravoxel-sized microscopy images. *BMC Bioinformatics* 13, 316. 10.1186/1471-2105-13-316 23181553 PMC3582611

[DEV199369C11] Bunce, C., Mckey, J. and Capel, B. (2021). Concerted morphogenesis of genital ridges and nephric ducts in the mouse captured through whole-embryo imaging. *Development* 148, dev199208. 10.1242/dev.199208 33795229 PMC8242465

[DEV199369C12] Cai, R., Pan, C., Ghasemigharagoz, A., Todorov, M. I., Förstera, B., Zhao, S., Bhatia, H. S., Parra-Damas, A., Mrowka, L., Theodorou, D. et al. (2019). Panoptic imaging of transparent mice reveals whole-body neuronal projections and skull–meninges connections. *Nat. Neurosci.* 22, 317-327. 10.1038/s41593-018-0301-3 30598527 PMC6494982

[DEV199369C13] Campbell-Thompson, M. and Tang, S.-C. (2021). Pancreas optical clearing and 3-D microscopy in health and diabetes. *Front. Endocrinol.* 12, 644826. 10.3389/fendo.2021.644826 PMC8108133 33981285

[DEV199369C14] Carrillo, M., Chuecos, M., Gandhi, K., Bednov, A., Moore, D. L., Maher, J., Ventolini, G., Ji, G. and Schlabritz-Loutsevitch, N. (2018). Optical tissue clearing in combination with perfusion and immunofluorescence for placental vascular imaging. *Medicine* 97, e12392. 10.1097/MD.0000000000012392 30278515 PMC6181621

[DEV199369C15] Chen, F., Tillberg, P. W. and Boyden, E. S. (2015). Expansion microscopy. *Science* 347, 543-548. 10.1126/science.1260088 25592419 PMC4312537

[DEV199369C16] Chi, J., Wu, Z., Choi, C. H. J., Nguyen, L., Tegegne, S., Ackerman, S. E., Crane, A., Marchildon, F., Tessier-Lavigne, M. and Cohen, P. (2018). Three-dimensional adipose tissue imaging reveals regional variation in beige fat biogenesis and PRDM16-dependent sympathetic neurite density. *Cell Metab.* 27, 226-236.e3. 10.1016/j.cmet.2017.12.011 29320703

[DEV199369C112] Choi, S. W., Guan, W. and Chung, K. (2021). Basic principles of hydrogel-based tissue transformation technologies and their applications. *Cell* 184, 4115-4136. 10.1016/j.cell.2021.07.009 34358468 PMC8372535

[DEV199369C17] Chung, K., Wallace, J., Kim, S.-Y., Kalyanasundaram, S., Andalman, A. S., Davidson, T. J., Mirzabekov, J. J., Zalocusky, K. A., Mattis, J., Denisin, A. K. et al. (2013). Structural and molecular interrogation of intact biological systems. *Nature* 497, 332-337. 10.1038/nature12107 23575631 PMC4092167

[DEV199369C18] Dekkers, J. F., Alieva, M., Wellens, L. M., Ariese, H. C. R., Jamieson, P. R., Vonk, A. M., Amatngalim, G. D., Hu, H., Oost, K. C., Snippert, H. J. G. et al. (2019). High-resolution 3D imaging of fixed and cleared organoids. *Nat. Protoc.* 14, 1756-1771. 10.1038/s41596-019-0160-8 31053799

[DEV199369C19] Dent, J. A., Polson, A. G. and Klymkowsky, M. W. (1989). A whole-mount immunocytochemical analysis of the expression of the intermediate filament protein vimentin in Xenopus. *Dev. Camb. Engl.* 105, 61-74. 10.1242/dev.105.1.61 2806118

[DEV199369C20] Dodt, H.-U., Leischner, U., Schierloh, A., Jährling, N., Mauch, C. P., Deininger, K., Deussing, J. M., Eder, M., Zieglgänsberger, W. and Becker, K. (2007). Ultramicroscopy: three-dimensional visualization of neuronal networks in the whole mouse brain. *Nat. Methods* 4, 331-336. 10.1038/nmeth1036 17384643

[DEV199369C21] Ertürk, A., Becker, K., Jährling, N., Mauch, C. P., Hojer, C. D., Egen, J. G., Hellal, F., Bradke, F., Sheng, M. and Dodt, H. U. (2012a). Three-dimensional imaging of solvent-cleared organs using 3DISCO. *Nat. Protoc.* 7, 1983-1995. 10.1038/nprot.2012.119 23060243

[DEV199369C22] Ertürk, A., Mauch, C. P., Hellal, F., Förstner, F., Keck, T., Becker, K., Jährling, N., Steffens, H., Richter, M., Hübener, M. et al. (2012b). Three-dimensional imaging of the unsectioned adult spinal cord to assess axon regeneration and glial responses after injury. *Nature Medicine* 18, 166-171. 10.1038/nm.2600 22198277

[DEV199369C23] Escalante, A., González-Martínez, R. and Herrera, E. (2020). New techniques for studying neurodevelopment. *Fac. Rev.* 9, 17. 10.12703/r/9-17 33659949 PMC7886075

[DEV199369C24] Felix, P. M., Gonçalves, A., Vicente, J. R., Fonseca, P. J., Amorim, M. C. P., Costa, J. L. and Martins, G. G. (2016). Optical micro-tomography “OPenT” allows the study of large toadfish Halobatrachus didactylus embryos and larvae. *Mech. Dev.* 140, 19-24. 10.1016/j.mod.2016.03.001 27000637

[DEV199369C25] Feuchtinger, A., Walch, A. and Dobosz, M. (2016). Deep tissue imaging: a review from a preclinical cancer research perspective. *Histochem. Cell Biol.* 146, 1-26. 10.1007/s00418-016-1495-7 27704211

[DEV199369C26] Friedmann, D., Pun, A., Adams, E. L., Lui, J. H., Kebschull, J. M., Grutzner, S. M., Castagnola, C., Tessier-Lavigne, M. and Luo, L. (2020). Mapping mesoscale axonal projections in the mouse brain using a 3D convolutional network. *Proc. Natl. Acad. Sci. USA* 117, 11068-11075. 10.1073/pnas.1918465117 32358193 PMC7245124

[DEV199369C27] Friocourt, F. and Chédotal, A. (2016). The Robo3 receptor, a key player in the development, evolution and function of commissural systems. *Dev. Neurobiol.* 77, 876-890. 10.1002/dneu.22478 28033646

[DEV199369C28] Friocourt, F., Lafont, A.-G., Kress, C., Pain, B., Manceau, M., Dufour, S. and Chédotal, A. (2017). Recurrent DCC gene losses during bird evolution. *Sci. Rep.* 7, 37569. 10.1038/srep37569 28240285 PMC5327424

[DEV199369C29] Friocourt, F., Kozulin, P., Belle, M., Suárez, R., Poï, N. D., Richards, L. J., Giacobini, P. and Chédotal, A. (2019). Shared and differential features of Robo3 expression pattern in amniotes. *J. Comp. Neurol.* 527, 2009-2029. 10.1002/cne.24648 30697732

[DEV199369C30] Gómez-Gaviro, M. V., Balaban, E., Bocancea, D., Lorrio, M. T., Pompeiano, M., Desco, M., Ripoll, J. and Vaquero, J. J. (2017). Optimized CUBIC protocol for three-dimensional imaging of chicken embryos at single-cell resolution. *Development* 144, 2092-2097. 10.1242/dev.145805 28432219

[DEV199369C31] Gómez-Gaviro, M. V., Sanderson, D., Ripoll, J. and Desco, M. (2020). Biomedical applications of tissue clearing and three-dimensional imaging in health and disease. *Iscience* 23, 101432. 10.1016/j.isci.2020.101432 32805648 PMC7452225

[DEV199369C32] Green, M. C. (1952). A rapid method for clearing and staining specimens for the demonstration of bone. *Ohio J. Sci.* 1, 31-33.

[DEV199369C33] Hahn, C., Becker, K., Saghafi, S., Pende, M., Avdibašić, A., Foroughipour, M., Heinz, D. E., Wotjak, C. T. and Dodt, H. (2019). High–resolution imaging of fluorescent whole mouse brains using stabilised organic media (sDISCO). *J. Biophotonics.* 12, e201800368. 10.1002/jbio.201800368 30932329

[DEV199369C34] Hama, H., Kurokawa, H., Kawano, H., Ando, R., Shimogori, T., Noda, H., Fukami, K., Sakaue-Sawano, A. and Miyawaki, A. (2011). Scale: a chemical approach for fluorescence imaging and reconstruction of transparent mouse brain. *Nat. Neurosci.* 14, 1481-1488. 10.1038/nn.2928 21878933

[DEV199369C35] Hallou, A., Yevick, H., Dumitrascu B. and Uhlmann V. (2021). Deep learning for bioimage analysis in developmental biology. *Development* 148, dev199616. 10.1242/dev.199616 34490888 PMC8451066

[DEV199369C36] Henning, Y., Osadnik, C. and Malkemper, E. P. (2018). EyeCi: Optical clearing and imaging of immunolabeled mouse eyes using light-sheet fluorescence microscopy. *Exp. Eye Res.* 180, 137-145. 10.1016/j.exer.2018.12.001 30578790

[DEV199369C37] Hörl, D., Rusak, F. R., Preusser, F., Tillberg, P., Randel, N., Chhetri, R. K., Cardona, A., Keller, P. J., Harz, H., Leonhardt, H. et al. (2019). BigStitcher: reconstructing high-resolution image datasets of cleared and expanded samples. *Nat. Methods* 16, 870-874. 10.1038/s41592-019-0501-0 31384047

[DEV199369C38] Hou, B., Zhang, D., Zhao, S., Wei, M., Yang, Z., Wang, S., Wang, J., Zhang, X., Liu, B., Fan, L. et al. (2015). Scalable and DiI-compatible optical clearance of the mammalian brain. *Front. Neuroanat.* 9, 19. 10.3389/fnana.2015.00019 25759641 PMC4338786

[DEV199369C39] Jacob, L., Boisserand, L. S. B., Geraldo, L. H. M., De Brito Neto, J., Mathivet, T., Antila, S., Barka, B., Xu, Y., Thomas, J.-M., Pestel, J. et al. (2019). Anatomy and function of the vertebral column lymphatic network in mice. *Nat. Commun.* 10, 4594-4516. 10.1038/s41467-019-12568-w 31597914 PMC6785564

[DEV199369C40] Jin, M., Nguyen, J. D., Weber, S. J., Mejias-Aponte, C. A., Madangopal, R. and Golden, S. A. (2019). SMART: An open source extension of WholeBrain for iDISCO+ LSFM intact mouse brain registration and segmentation. *bioRxiv* 727529. 10.1101/727529. PMC9070730 35396258

[DEV199369C41] Jing, D., Zhang, S., Luo, W., Gao, X., Men, Y., Ma, C., Liu, X., Yi, Y., Bugde, A., Zhou, B. O. et al. (2018). Tissue clearing of both hard and soft tissue organs with the PEGASOS method. *Cell Res.* 28, 803-818. 10.1038/s41422-018-0049-z 29844583 PMC6082844

[DEV199369C42] Kagami, K., Shinmyo, Y., Ono, M., Kawasaki, H. and Fujiwara, H. (2017). Three-dimensional visualization of intrauterine conceptus through the uterine wall by tissue clearing method. *Sci. Rep.* 7, 5964. 10.1038/s41598-017-06549-6 28729622 PMC5519638

[DEV199369C43] Ke, M.-T., Fujimoto, S. and Imai, T. (2013). SeeDB: a simple and morphology-preserving optical clearing agent for neuronal circuit reconstruction. *Nat. Neurosci.* 16, 1154-1161. 10.1038/nn.3447 23792946

[DEV199369C44] Ke, M.-T., Nakai, Y., Fujimoto, S., Takayama, R., Yoshida, S., Kitajima, T. S., Sato, M. and Imai, T. (2016). Super-resolution mapping of neuronal circuitry with an index-optimized clearing agent. *Cell Reports* 14, 2718-2732. 10.1016/j.celrep.2016.02.057 26972009

[DEV199369C45] Kim, S.-Y., Cho, J. H., Murray, E., Bakh, N., Choi, H., Ohn, K., Ruelas, L., Hubbert, A., Mccue, M., Vassallo, S. L. et al. (2015). Stochastic electrotransport selectively enhances the transport of highly electromobile molecules. *Proc. Natl. Acad. Sci. USA* 112, E6274-E6283. 10.1073/pnas.1510133112 26578787 PMC4655572

[DEV199369C46] Kirst, C., Skriabine, S., Vieites-Prado, A., Topilko, T., Bertin, P., Gerschenfeld, G., Verny, F., Topilko, P., Michalski, N., Tessier-Lavigne, M. et al. (2020). Mapping the fine-scale organization and plasticity of the brain vasculature. *Cell* 180, 780-795.e25. 10.1016/j.cell.2020.01.028 32059781

[DEV199369C47] Klingberg, A., Hasenberg, A., Ludwig-Portugall, I., Medyukhina, A., Männ, L., Brenzel, A., Engel, D. R., Figge, M. T., Kurts, C. and Gunzer, M. (2017). Fully automated evaluation of total glomerular number and capillary tuft size in nephritic kidneys using lightsheet microscopy. *J. Am. Soc. Nephrol.* 28, 452-459. 10.1681/ASN.2016020232 27487796 PMC5280021

[DEV199369C48] Kolesová, H., Bartoš, M., Hsieh, W. C., Olejníčková, V. and Sedmera, D. (2018). Novel approaches to study coronary vasculature development in mice. *Dev. Dyn.* 247, 1018-1027. 10.1002/dvdy.24637 29770532

[DEV199369C49] Kolesová, H., Olejníčková, V., Kvasilová, A., Gregorovičová, M. and Sedmera, D. (2021). Tissue clearing and imaging methods for cardiovascular development. *Iscience* 24, 102387. 10.1016/j.isci.2021.102387 33981974 PMC8086021

[DEV199369C50] Konno, A. and Okazaki, S. (2018). Aqueous-based tissue clearing in crustaceans. *Zoological. Lett.* 4, 13. 10.1186/s40851-018-0099-6 29930867 PMC5991465

[DEV199369C51] Ku, T., Guan, W., Evans, N. B., Sohn, C. H., Albanese, A., Kim, J.-G., Frosch, M. P. and Chung, K. (2020). Elasticizing tissues for reversible shape transformation and accelerated molecular labeling. *Nat. Methods* 17, 609-613. 10.1038/s41592-020-0823-y 32424271 PMC8056749

[DEV199369C52] Kurihara, D., Mizuta, Y., Sato, Y. and Higashiyama, T. (2015). ClearSee: a rapid optical clearing reagent for whole-plant fluorescence imaging. *Development* 142, 4168-4179. 10.1242/dev.127613 26493404 PMC4712841

[DEV199369C53] Kuwajima, T., Sitko, A. A., Bhansali, P., Jurgens, C., Guido, W. and Mason, C. (2013). ClearT: a detergent- and solvent-free clearing method for neuronal and non-neuronal tissue. *Development* 140, 1364-1368. 10.1242/dev.091844 23444362 PMC3912244

[DEV199369C54] Masselink, W., Reumann, D., Murawala, P., Pasierbek, P., Taniguchi, Y., Bonnay, F., Meixner, K., Knoblich, J. A. and Tanaka, E. M. (2019). Broad applicability of a streamlined ethyl cinnamate-based clearing procedure. *Development* 146, dev166884. 10.1242/dev.166884 30665888 PMC7115989

[DEV199369C55] Molbay, M., Kolabas, Z. I., Todorov, M. I., Ohn, T. and Ertürk, A. (2021). A guidebook for DISCO tissue clearing. *Mol. Syst. Biol.* 17, e9807. 10.15252/msb.20209807 33769689 PMC7995442

[DEV199369C56] Murakami, T. C., Mano, T., Saikawa, S., Horiguchi, S. A., Shigeta, D., Baba, K., Sekiya, H., Shimizu, Y., Tanaka, K. F., Kiyonari, H. et al. (2018). A three-dimensional single-cell-resolution whole-brain atlas using CUBIC-X expansion microscopy and tissue clearing. *Nat. Neurosci.* 21, 625-637. 10.1038/s41593-018-0109-1 29507408

[DEV199369C57] Murray, E., Cho, J. H., Goodwin, D., Ku, T., Swaney, J., Kim, S.-Y., Choi, H., Park, Y.-G., Park, J.-Y., Hubbert, A. et al. (2015). Simple, scalable proteomic imaging for high-dimensional profiling of intact systems. *Cell* 163, 1500-1514. 10.1016/j.cell.2015.11.025 26638076 PMC5275966

[DEV199369C58] Palmer, W. M., Martin, A. P., Flynn, J. R., Reed, S. L., White, R. G., Furbank, R. T. and Grof, C. P. L. (2015). PEA-CLARITY: 3D molecular imaging of whole plant organs. *Sci. Rep.* 5, 13492. 10.1038/srep13492 26328508 PMC4556961

[DEV199369C59] Pan, C., Cai, R., Quacquarelli, F. P., Ghasemigharagoz, A., Lourbopoulos, A., Matryba, P., Plesnila, N., Dichgans, M., Hellal, F. and Ertürk, A. (2016). Shrinkage-mediated imaging of entire organs and organisms using uDISCO. *Nat. Methods* 13, 859-867. 10.1038/nmeth.3964 27548807

[DEV199369C60] Pan, C., Schoppe, O., Parra-Damas, A., Cai, R., Todorov, M. I., Gondi, G., Von Neubeck, B., Böğürcü-Seidel, N., Seidel, S. et al. (2019). Deep learning reveals cancer metastasis and therapeutic antibody targeting in the entire body. *Cell* 179, 1661-1676.e19. 10.1016/j.cell.2019.11.013 31835038 PMC7591821

[DEV199369C61] Park, Y.-G., Sohn, C. H., Chen, R., Mccue, M., Yun, D. H., Drummond, G. T., Ku, T., Evans, N. B., Oak, H. C., Trieu, W. et al. (2018). Protection of tissue physicochemical properties using polyfunctional crosslinkers. *Nat. Biotechnol.* [Epub ahead of print]. 10.1038/nbt.4281 PMC6579717 30556815

[DEV199369C62] Pende, M., Becker, K., Wanis, M., Saghafi, S., Kaur, R., Hahn, C., Pende, N., Foroughipour, M., Hummel, T. and Dodt, H.-U. (2018). High-resolution ultramicroscopy of the developing and adult nervous system in optically cleared Drosophila melanogaster. *Nat. Commun.* 9, 4731. 10.1038/s41467-018-07192-z 30413688 PMC6226481

[DEV199369C63] Pende, M., Vadiwala, K., Schmidbaur, H., Stockinger, A. W., Murawala, P., Saghafi, S., Dekens, M. P. S., Becker, K., Revilla-I-Domingo, R., Papadopoulos, S.-C. et al. (2020). A versatile depigmentation, clearing, and labeling method for exploring nervous system diversity. *Sci. Adv.* 6, eaba0365. 10.1126/sciadv.aba0365 32523996 PMC7259959

[DEV199369C64] Perin, P., Voigt, F. F., Bethge, P., Helmchen, F. and Pizzala, R. (2019). iDISCO+ for the study of neuroimmune architecture of the rat auditory brainstem. *Front. Neuroanat.* 13, 15. 10.3389/fnana.2019.00015 30814937 PMC6381022

[DEV199369C65] Qi, Y., Yu, T., Xu, J., Wan, P., Ma, Y., Zhu, J., Li, Y., Gong, H., Luo, Q. and Zhu, D. (2019). FDISCO: Advanced solvent-based clearing method for imaging whole organs. *Sci. Adv.* 5, eaau8355. 10.1126/sciadv.aau8355 30746463 PMC6357753

[DEV199369C66] Renier, N., Wu, Z., Simon, D. J., Yang, J., Ariel, P. and Tessier-Lavigne, M. (2014). iDISCO: a simple, rapid method to immunolabel large tissue samples for volume imaging. *Cell* 159, 896-910. 10.1016/j.cell.2014.10.010 25417164

[DEV199369C67] Renier, N., Adams, E. L., Kirst, C., Wu, Z., Azevedo, R., Kohl, J., Autry, A. E., Kadiri, L., Venkataraju, K. U., Zhou, Y. et al. (2016). Mapping of brain activity by automated volume analysis of immediate early genes. *Cell* 165, 1789-1802. 10.1016/j.cell.2016.05.007 27238021 PMC4912438

[DEV199369C68] Richardson, D. S. and Lichtman, J. W. (2015). Clarifying tissue clearing. *Cell* 162, 246-257. 10.1016/j.cell.2015.06.067 26186186 PMC4537058

[DEV199369C69] Richardson, L., Vargas, G., Brown, T., Ochoa, L., Trivedi, J., Kacerovský, M., Lappas, M. and Menon, R. (2017). Redefining 3Dimensional placental membrane microarchitecture using multiphoton microscopy and optical clearing. *Placenta* 53, 66-75. 10.1016/j.placenta.2017.03.017 28487023

[DEV199369C70] Schmidt, E. R. E., Brignani, S., Adolfs, Y., Lemstra, S., Demmers, J., Vidaki, M., Donahoo, A.-L. S., Lilleväli, K., Vasar, E., Richards, L. J. et al. (2014). Subdomain-mediated axon-axon signaling and chemoattraction cooperate to regulate afferent innervation of the lateral habenula. *Neuron* 83, 372-387. 10.1016/j.neuron.2014.05.036 25033181

[DEV199369C71] Schwarz, M. K., Scherbarth, A., Sprengel, R., Engelhardt, J., Theer, P. and Giese, G. (2015). Fluorescent-protein stabilization and high-resolution imaging of cleared, intact mouse brains. *PLoS ONE* 10, e0124650. 10.1371/journal.pone.0124650 25993380 PMC4439039

[DEV199369C72] Serizawa, T., Isotani, A., Matsumura, T., Nakanishi, K., Nonaka, S., Shibata, S., Ikawa, M. and Okano, H. (2019). Developmental analyses of mouse embryos and adults using a non-overlapping tracing system for all three germ layers. *Development* 146, dev174938. 10.1242/dev.174938 31597657

[DEV199369C73] Shamonin, D. P., Bron, E. E., Lelieveldt, B. P. F., Smits, M., Klein, S. and Staring, M. (2014). Fast parallel image registration on CPU and GPU for diagnostic classification of Alzheimer's disease. *Frontiers in Neuroinformatics* 7, 50. 24474917 10.3389/fninf.2013.00050 PMC3893567

[DEV199369C74] Sommer, C., Straehle, C., Kothe, U. and Hamprecht, F. A. (2011). Ilastik: interactive learning and segmentation toolkit. *2011 Ieee Int Symposium Biomed Imaging Nano Macro* 1, 230-233. 10.1109/ISBI.2011.5872394

[DEV199369C75] Spalteholz, W. (1911). A Method for the Clearing of Human and Animal Specimens. S. Hirzel.

[DEV199369C76] Spalteholz, W. (1914). Über das Durchsichtigmachen von menschlichen und tierischen Präparaten und seine theoretischen Bedingungen, nebst Anhang: Über Knochenfärbung. S. Hirzel.

[DEV199369C77] Stewart, T. A., Hughes, K., Hume, D. A. and Davis, F. M. (2019). Developmental stage-specific distribution of macrophages in mouse mammary gland. *Front. Cell Dev. Biol.* 7, 250. 10.3389/fcell.2019.00250 31709255 PMC6821639

[DEV199369C78] Susaki, E. A. and Ueda, H. R. (2016). Whole-body and whole-organ clearing and imaging techniques with single-cell resolution: toward organism-level systems biology in mammals. *Cell Chem. Biol.* 23, 137-157. 10.1016/j.chembiol.2015.11.009 26933741

[DEV199369C79] Susaki, E. A., Tainaka, K., Perrin, D., Kishino, F., Tawara, T., Watanabe, T. M., Yokoyama, C., Onoe, H., Eguchi, M., Yamaguchi, S. et al. (2014). Whole-brain imaging with single-cell resolution using chemical cocktails and computational analysis. *Cell* 157, 726-739. 10.1016/j.cell.2014.03.042 24746791

[DEV199369C80] Susaki, E. A., Tainaka, K., Perrin, D., Yukinaga, H., Kuno, A. and Ueda, H. R. (2015). Advanced CUBIC protocols for whole-brain and whole-body clearing and imaging. *Nat. Protoc.* 10, 1709-1727. 10.1038/nprot.2015.085 26448360

[DEV199369C81] Susaki, E. A., Shimizu, C., Kuno, A., Tainaka, K., Li, X., Nishi, K., Morishima, K., Ono, H., Ode, K. L., Saeki, Y. et al. (2020). Versatile whole-organ/body staining and imaging based on electrolyte-gel properties of biological tissues. *Nat. Commun.* 11, 1982. 10.1038/s41467-020-15906-5 32341345 PMC7184626

[DEV199369C82] Tainaka, K., Kubota, S. I., Suyama, T. Q., Susaki, E. A., Perrin, D., Ukai-Tadenuma, M., Ukai, H. and Ueda, H. R. (2014). Whole-body imaging with single-cell resolution by tissue decolorization. *Cell* 159, 911-924. 10.1016/j.cell.2014.10.034 25417165

[DEV199369C83] Tainaka, K., Kuno, A., Kubota, S. I., Murakami, T. and Ueda, H. R. (2016). Chemical principles in tissue clearing and staining protocols for whole-body cell profiling. *Annu. Rev. Cell Dev. Biol.* 32, 713-741. 10.1146/annurev-cellbio-111315-125001 27298088

[DEV199369C84] Tainaka, K., Murakami, T. C., Susaki, E. A., Shimizu, C., Saito, R., Takahashi, K., Hayashi-Takagi, A., Sekiya, H., Arima, Y., Nojima, S. et al. (2018). Chemical landscape for tissue clearing based on hydrophilic reagents. *Cell Reports* 24, 2196-2210.e9. 10.1016/j.celrep.2018.07.056 30134179

[DEV199369C85] Tischfield, M. A., Baris, H. N., Wu, C., Rudolph, G., Van Maldergem, L., He, W., Chan, W.-M., Andrews, C., Demer, J. L. et al. (2010). Human TUBB3 mutations perturb microtubule dynamics, kinesin interactions, and axon guidance. *Cell* 140, 74-87. 10.1016/j.cell.2009.12.011 20074521 PMC3164117

[DEV199369C86] Todorov, M. I., Paetzold, J. C., Schoppe, O., Tetteh, G., Shit, S., Efremov, V., Todorov-Völgyi, K., Düring, M., Dichgans, M., Piraud, M. et al. (2020). Machine learning analysis of whole mouse brain vasculature. *Nat. Methods* 17, 442-449. 10.1038/s41592-020-0792-1 32161395 PMC7591801

[DEV199369C87] Treweek, J. B., Chan, K. Y., Flytzanis, N. C., Yang, B., Deverman, B. E., Greenbaum, A., Lignell, A., Xiao, C., Cai, L., Ladinsky, M. S. et al. (2015). Whole-body tissue stabilization and selective extractions via tissue-hydrogel hybrids for high-resolution intact circuit mapping and phenotyping. *Nat. Protoc.* 10, 1860-1896. 10.1038/nprot.2015.122 26492141 PMC4917295

[DEV199369C88] Ueda, H. R., Dodt, H.-U., Osten, P., Economo, M. N., Chandrashekar, J. and Keller, P. J. (2020a). Whole-brain profiling of cells and circuits in mammals by tissue clearing and light-sheet microscopy. *Neuron* 106, 369-387. 10.1016/j.neuron.2020.03.004 32380050 PMC7213014

[DEV199369C89] Ueda, H. R., Ertürk, A., Chung, K., Gradinaru, V., Chédotal, A., Tomancak, P. and Keller, P. J. (2020b). Tissue clearing and its applications in neuroscience. *Nat. Rev. Neurosci.* 21, 61-79. 10.1038/s41583-019-0250-1. 31896771 PMC8121164

[DEV199369C90] Vigouroux, R. J., Belle, M. and Chédotal, A. (2017). Neuroscience in the third dimension: shedding new light on the brain with tissue clearing. *Mol. Brain* 10, 33. 10.1186/s13041-017-0314-y 28728585 PMC5520295

[DEV199369C91] Vigouroux, R. J., Cesar, Q., Chédotal, A. and Nguyen-Ba-Charvet, K. T. (2020). Revisiting the role of Dcc in visual system development with a novel eye clearing method. *Elife* 9, e51275. 10.7554/eLife.51275 32096760 PMC7062470

[DEV199369C92] Vigouroux, R. J., Duroure, K., Vougny, J., Albadri, S., Kozulin, P., Herrera, E., Nguyen-Ba-Charvet, K., Braasch, I., Suárez, R., Bene, F. D. et al. (2021). Bilateral visual projections exist in non-teleost bony fish and predate the emergence of tetrapods. *Science* 372, 150-156. 10.1126/science.abe7790 33833117 PMC9576344

[DEV199369C93] Voigt, F. F., Kirschenbaum, D., Platonova, E., Pagès, S., Campbell, R. A. A., Kästli, R., Schaettin, M., Egolf, L., Van Der Bourg, A. et al. (2019). The mesoSPIM initiative: open-source light-sheet microscopes for imaging cleared tissue. *Nat. Methods* 162, 246-244. 10.1038/s41592-019-0554-0 PMC6824906 31527839

[DEV199369C94] Wang, Q., Ding, S.-L., Li, Y., Royall, J., Feng, D., Lesnar, P., Graddis, N., Naeemi, M., Facer, B., Ho, A. et al. (2020). The Allen mouse brain common coordinate framework: a 3D reference atlas. *Cell* 181, 936-953.e20. 10.1016/j.cell.2020.04.007 32386544 PMC8152789

[DEV199369C95] Wang, X., Zeng, W., Yang, X., Fang, C., Han, Y. and Fei, P. (2021). Bi-channel Image Registration and Deep-learning Segmentation (BIRDS) for efficient, versatile 3D mapping of mouse brain. *Elife* 10, e63455. 10.7554/eLife.63455 33459255 PMC7840180

[DEV199369C96] Wassie, A. T., Zhao, Y. and Boyden, E. S. (2019). Expansion microscopy: principles and uses in biological research. *Nat. Methods* 16, 33-41. 10.1038/s41592-018-0219-4 30573813 PMC6373868

[DEV199369C97] Watanabe, T., Nakamura, R., Takase, Y., Susaki, E. A., Ueda, H. R., Tadokoro, R. and Takahashi, Y. (2018). Comparison of the 3-D patterns of the parasympathetic nervous system in the lung at late developmental stages between mouse and chicken. *Dev. Biol.* 444, S325-S336. 10.1016/j.ydbio.2018.05.014 29792856

[DEV199369C98] Weisblat, D. A. and Kuo, D.-H. (2009). Whole-mount preparation of helobdella (Leech) embryos for microscopy. *Cold Spring Harb. Protoc.* 2009, pdb.prot5195. 10.1101/pdb.prot5195 20147138

[DEV199369C99] Weiss, K. R., Voigt, F. F., Shepherd, D. P. and Huisken, J. (2021). Tutorial: practical considerations for tissue clearing and imaging. *Nat. Protoc.* 16, 2732-2748. 10.1038/s41596-021-00502-8 34021294 PMC10542857

[DEV199369C100] Woo, J., Kang, H., Lee, E. Y., Park, S. and Cho, Y. E. (2020). Investigation of PRDM7 and PRDM12 expression pattern during mouse embryonic development by using a modified passive clearing technique. *Biochem. Bioph. Res. Commun.* 524, 346-353. 10.1016/j.bbrc.2019.12.133 32000999

[DEV199369C101] Xu, F., Shen, Y., Ding, L., Yang, C.-Y., Tan, H., Wang, H., Zhu, Q., Xu, R., Wu, F., Xiao, Y. et al. (2021). High-throughput mapping of a whole rhesus monkey brain at micrometer resolution. *Nat. Biotechnol.* [Epub ahead of print]. 10.1038/s41587-021-00986-5 34312500

[DEV199369C102] Yang, B., Treweek, J. B., Kulkarni, R. P., Deverman, B. E., Chen, C.-K., Lubeck, E., Shah, S., Cai, L. and Gradinaru, V. (2014). Single-cell phenotyping within transparent intact tissue through whole-body clearing. *Cell* 158, 945-958. 10.1016/j.cell.2014.07.017 25088144 PMC4153367

[DEV199369C103] Ye, L., Allen, W. E., Thompson, K. R., Tian, Q., Hsueh, B., Ramakrishnan, C., Wang, A.-C., Jennings, J. H., Adhikari, A., Halpern, C. H. et al. (2016). Wiring and molecular features of prefrontal ensembles representing distinct experiences. *Cell* 165, 1776-1788. 10.1016/j.cell.2016.05.010 27238022 PMC5708551

[DEV199369C104] Young, D. M., Duhn, C., Gilson, M., Nojima, M., Yuruk, D., Kumar, A., Yu, W. and Sanders, S. J. (2020). Whole brain image analysis and anatomical atlas 3D generation using magellanmapper. *Curr. Protoc. Neurosci.* 94, e104. 10.1002/cpns.104 32981139 PMC7781073

[DEV199369C105] Young, D. M., Darbandi, S. F., Schwartz, G., Bonzell, Z., Yuruk, D., Nojima, M., Gole, L. C., Rubenstein, J. L., Yu, W. and Sanders, S. J. (2021). Constructing and optimizing 3D atlases from 2D data with application to the developing mouse brain. *Elife* 10, e61408. 10.7554/eLife.61408 33570495 PMC7994002

[DEV199369C106] Yu, T., Zhu, J., Li, Y., Ma, Y., Wang, J., Cheng, X., Jin, S., Sun, Q., Li, X., Gong, H. et al. (2018). RTF: a rapid and versatile tissue optical clearing method. *Sci. Rep.* 8, 1964. 10.1038/s41598-018-20306-3 29386656 PMC5792593

[DEV199369C107] Yu, T., Zhu, J., Li, D. and Zhu, D. (2021). Physical and chemical mechanisms of tissue optical clearing. *Iscience* 24, 102178. 10.1016/j.isci.2021.102178 33718830 PMC7920833

[DEV199369C108] Yuan, J., Deng, W., Cha, J., Sun, X., Borg, J.-P. and Sudhansu, K. D. (2018). Tridimensional visualization reveals direct communication between the embryo and glands critical for implantation. *Nat. Commun.* 9, 603. 10.1038/s41467-018-03092-4 29426931 PMC5807548

[DEV199369C109] Yun, D. H., Park, Y.-G., Cho, J. H., Kamentsky, L., Evans, N. B., Albanese, A., Xie, K., Swaney, J., Sohn, C. H., Tian, Y. et al. (2019). Ultrafast immunostaining of organ-scale tissues for scalable proteomic phenotyping. *bioRxiv* 660373. 10.1101/660373

[DEV199369C110] Zhao, S., Todorov, M. I., Cai, R., -Maskari, R. A., Steinke, H., Kemter, E., Mai, H., Rong, Z., Warmer, M., Stanic, K. et al. (2020). Cellular and molecular probing of intact human organs. *Cell* 180, 796-812.e19. 10.1016/j.cell.2020.01.030 32059778 PMC7557154

[DEV199369C111] Zucker, R. M., Hunter, S. and Rogers, J. M. (1998). Confocal laser scanning microscopy of apoptosis in organogenesis–stage mouse embryos. *Cytometry* 33, 348-354. 10.1002/(SICI)1097-0320(19981101)33:3<348::AID-CYTO9>3.0.CO;2-C 9822346

